# Roussoelins A and B: two phenols with antioxidant capacity from ascidian-derived fungus *Roussoella siamensis* SYSU-MS4723

**DOI:** 10.1007/s42995-020-00066-8

**Published:** 2020-10-23

**Authors:** Senhua Chen, Hongjie Shen, Yanlian Deng, Heng Guo, Minghua Jiang, Zhenger Wu, Huimin Yin, Lan Liu

**Affiliations:** 1 School of Marine Sciences, Sun Yat-Sen University, Guangzhou, 510006 China; 2 School of Pharmacy, Guangdong Medical University, Dongguan, 523808 China; 3 Southern Laboratory of Ocean Science and Engineering (Guangdong, Zhuhai), Zhuhai, 519000 China

**Keywords:** Phenols, Antioxidant capacity, Ascidian-derived fungus, *Roussoella siamensis*

## Abstract

Ascidian-derived microorganisms are a significant source of pharmacologically active metabolites with interesting structural properties. When discovering bioactive molecules from ascidian-derived fungi, two new phenols, roussoelins A (**1**) and B (**2**), and ten known polyketides (**3**–**12**) were isolated from the ascidian-derived fungus *Roussoella siamensis* SYSU-MS4723. The planar structure of compounds **1** and **2** was established by analysis of HR-ESIMS and NMR data. The conformational analysis of the new compounds was assigned according to coupling constants and selective gradient NOESY experiments, and absolute configurations were completed by the modified Mosher’s method. Among the isolated compounds, **1**, **2**, and **9** showed moderate antioxidant capacity.

**Graphical abstract:**

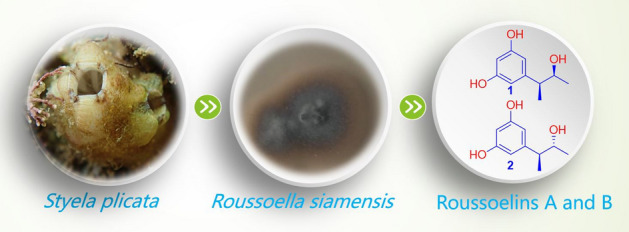

**Electronic supplementary material:**

The online version of this article (10.1007/s42995-020-00066-8) contains supplementary material, which is available to authorized users.

## Introduction

Marine organisms have been a significant natural source for the discovery of multiple pharmacologically active molecules with various structures (Blunt et al. 2017, 2018; Carroll et al. 2019; Jiang et al. 2020; Liu et al. 2019). Among them, about 150 molecules with a wide range of bioactivities have been discovered from ascidian-derived microorganisms (Bugni and Ireland 2004; Chen et al. 2018; Donia et al. 2006). For instance, the lomaiviticins A and B with an intricate dimeric diazobenzofluorene glycoside structure and antitumor activity were discovered from ascidian-derived Actinomycetes *Micromonospora lomaivitiensis* (He et al. 2001). The ascidian-associated fungus *Eurotiomycetes* strain 110,162 produced an anti-mycobacterial oxazinin A that contained a unique dimeric structure (Lin et al. 2014b). Another ascidian-derived fungus *Trichobotrys effuse* 4729 yielded an anti-glioma trichobamide A that was a pyrrocidine alkaloid containing a novel tetrahydro-5*H*-furo[2,3-b]pyrrol-5-one moiety (Chen et al. 2019b).

Since the first report in 1997 from Crews’ research group describing the chemical investigation of a fungus *Pithomyces* sp. (isolated from the Indo-Pacific tunicate *Oxycorynia fascicularis*) to afford polekeides (pitholides A–D) (Wang et al. 1997), a total of 52 new metabolites have been reported from 22 research papers involved in ascidian-derived fungi (Belofsky et al. 2000; Bugni et al. 2000; Chen et al. 2019a, b; Dewapriya et al. 2017, 2018; Garo et al. 2003; Ivanets et al. 2018; Li et al. 2020; Lin et al. 2014a; MalmstrøM et al. 2000; Montenegro et al. 2012; Motohashi et al. 2009; Murshid et al. 2016; Niaz et al. 2019; Shaala and Youssef 2015; Smetanina et al. 2004; Song et al. 2019; Sumilat et al. 2017; Xin et al. 2007; Yamazaki et al. 2015; Yurchenko et al. 2017). There were 21 strains (including one strain of unidentified fungus) belonging to eight genera (*Acremonium*, *Aspergillus*, *Humicola*, *Penicillium*, *Pithomyces*, *Talaromyces*, *Trichobotrys*, and *Trichoderma*). *Penicillium* (34.6%, 18) and *Aspergillus* (28.8%, 15) each represents more than 25% of the total and are the dominant producers of new metabolites, whose contributions together comprise more than half of the total. These new metabolites with various structures (including polyketide, alkaloid, sesquiterpene, merosesquiterpene, peptide, cerebroside) displayed numerous biological activities, including cytotoxicity (Chen et al. 2019b), antibacterial activity (Dewapriya et al. 2018), antifungal activity (Murshid et al. 2016), anti-inflammatory activity (Belofsky et al. 2000; Chen et al. 2019a), enzyme inhibitor activity (Yamazaki et al. 2015), and other activities (Lin et al. 2014a).

**Fig. 1:**
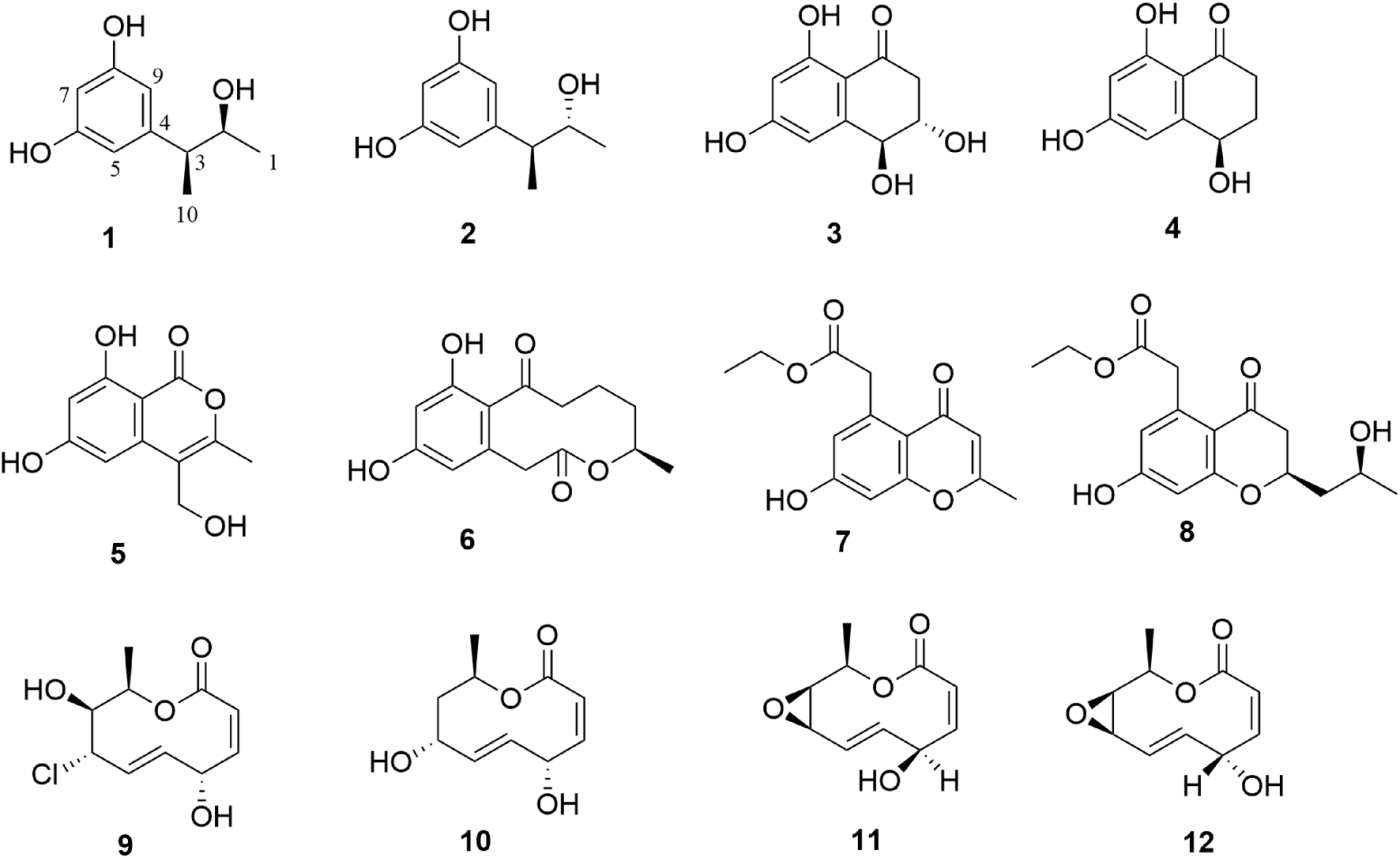
Chemical structures of **1**–**12**

Though 25 genera fungi of 19 families in two phyla have been derived from the ascidian, eight genera have been chemically investigated and the number of reports describing natural products from ascidian-derived fungi is still low. Recently, we focused on bioactive secondary metabolites from ascidian-derived fungi isolated from the South China Sea (Chen et al. 2019a, b; Niaz et al. 2019). As we continue to discover bioactive molecules from ascidian-derived fungi, two new 5-(3-hydroxybutan-2-yl)benzene-1,3-diol, roussoelins A (**1**) and B (**2**), together with ten known polyketides (**3**–**12**) were obtained from the ascidian-derived fungus *Roussoella siamensis* SYSU-MS4723 (Fig. 1), whose secondary metabolites were studied for the first time from a genus of an ascidian-derived fungi. The conformational analysis was assigned according to coupling constants and selective gradient NOESY experiments, and absolute configurations were finally identified by a modified version of Mosher’s method (Ohtani et al. 1991). The cytotoxicity, anti-inflammatory, and antioxidant activity of these molecules are reported herein.

## Results and discussion

The EtOAc extract of *R. sia*mensis SYSU-MS4723 was subjected to repeated silica gel and Sephadex LH-20 column chromatography, followed by semipreparative HPLC, to afford two new phenols, roussoelins A (**1**) and B (**2**), and ten known polyketides (**3**–**12**).

**Fig. 2:**
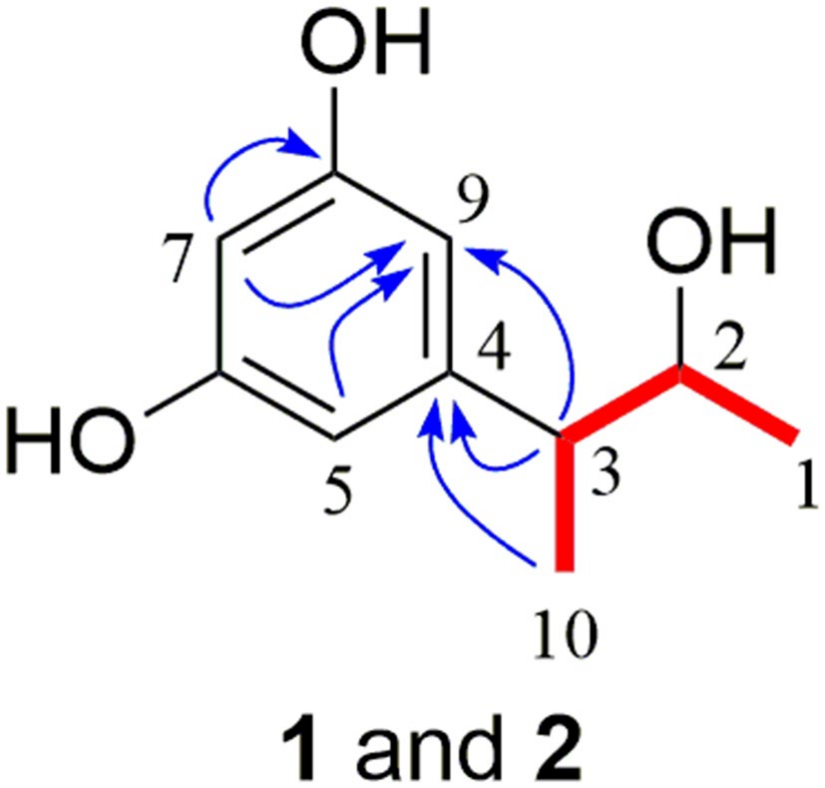
Key ^1^H-^1^H COSY (red line) and HMBC (blue arrow) correlations of compounds **1** and **2** (color figure online)

Roussoelin A (**1**) was isolated as a colorless oil. The molecular formula C_10_H_14_O_3_ was assigned by the negative HR-ESIMS ions at *m*/*z* 181.08712 [M^−^H]^−^ (calcd. for C_10_H_13_O_3_, 181.08702) (Supplementary Fig. S1), indicating four degrees of unsaturation. The IR spectrum (Supplementary Fig. S2) of **1** revealed the presence of a hydroxy (3346 cm^−1^) group. The ^1^H NMR data (Supplementary Fig. S3) (Table 1) revealed three aromatic protons [*δ*_H_ 6.13 (2H, *d*, *J* = 2.2 Hz); 6.10 (1H, *t*, *J* = 2.2 Hz)], indicating a 1,3,5-trisubstituted aromatic ring; two methyls [*δ*_H_ 3.69 (1H, dq, *J* = 8.3, 6.3 Hz); 2.39 (1H, m)]; and two methyl groups [*δ*_H_ 1.00 (3H, *d*, *J* = 6.3 Hz); 1.26(1H, *t*, *J* = 6.9 Hz)]. The ^13^C NMR (Supplementary Fig. S4) and HSQC data (Table 1) of **1** showed the presence of 10 carbons. Among them, six sp^2^ hybridized carbons (*δ*_C_ 101.5, 107.2, 107.2, 148.8, 159.4, 159.4) belonged to a benzene ring, while there were four remaining sp^3^ hybridized carbons, one of them (*δ*_C_ 73.4) directly connected with a heteroatom. The planar structure of **1** was mainly identified by ^1^H-^1^H COSY (Supplementary Fig. S5), HSQC (Supplementary Fig. S6), and HMBC (Supplementary Fig. S7) spectra (Fig. 2). A 3-hydroxybutan-2-yl group was deduced by the ^1^H-^1^H COSY cross peak between H-1 and H-2, H-2 and H-3, H-3 and H-10, together with HMBC correlations from H-1 to C-2 and C-3, H-10 to C-2 and C-3. Key HMBC correlations from H-10 and H-3 to C-4 suggested that the 3-hydroxybutan-2-yl group was linked to C-4 of an aromatic ring. Two hydroxyl groups were located on C-6 (*δ*_C_ 159.4) and C-8 (*δ*_C_ 159.4) of an aromatic ring according to the chemical shift and the HMBC correlations from H-7 to C-6 and C-8. The planar structure of **1** was elucidated as 5-(3-hydroxybutan-2-yl)benzene-1,3-diol.

**Table 1:** ^1^H (400 MHz) and ^13^C (100 MHz) NMR spectroscopic data for compounds **1** and **2** in CD_3_OD

No.	**1**		**2**	
	*δ*_C_, type	*δ*_H_, mult (*J* in Hz)	*δ*_C_, type	*δ*_H_, mult (*J* in Hz)
1	22.0, CH_3_	1.00, *d* (6.3)	20.1, CH_3_	1.10, *d* (7.3)
2	73.4, CH	3.69, dq (8.3, 6.3)	72.8, CH	3.82, *p* (6.3)
3	49.6, CH	2.39, m	48.4, CH	2.58, *p* (7.0)
4	148.8, C		147.9, C	
5	107.2, CH	6.13, *d* (2.2)	107.7, CH	6.20, *d* (2.2)
6	159.4, C		159.2, C	
7	101.5, CH	6.10, *t* (2.2)	101.5, CH	6.10, *t* (2.2)
8	159.4, C		159.2, C	
9	107.2, CH	6.13, *d* (2.1)	107.7, CH	6.20, *d* (2.2)
10	18.7, CH_3_	1.26, *d* (6.9)	16.9, CH_3_	1.18, *d* (7.1)

**Fig. 3:**
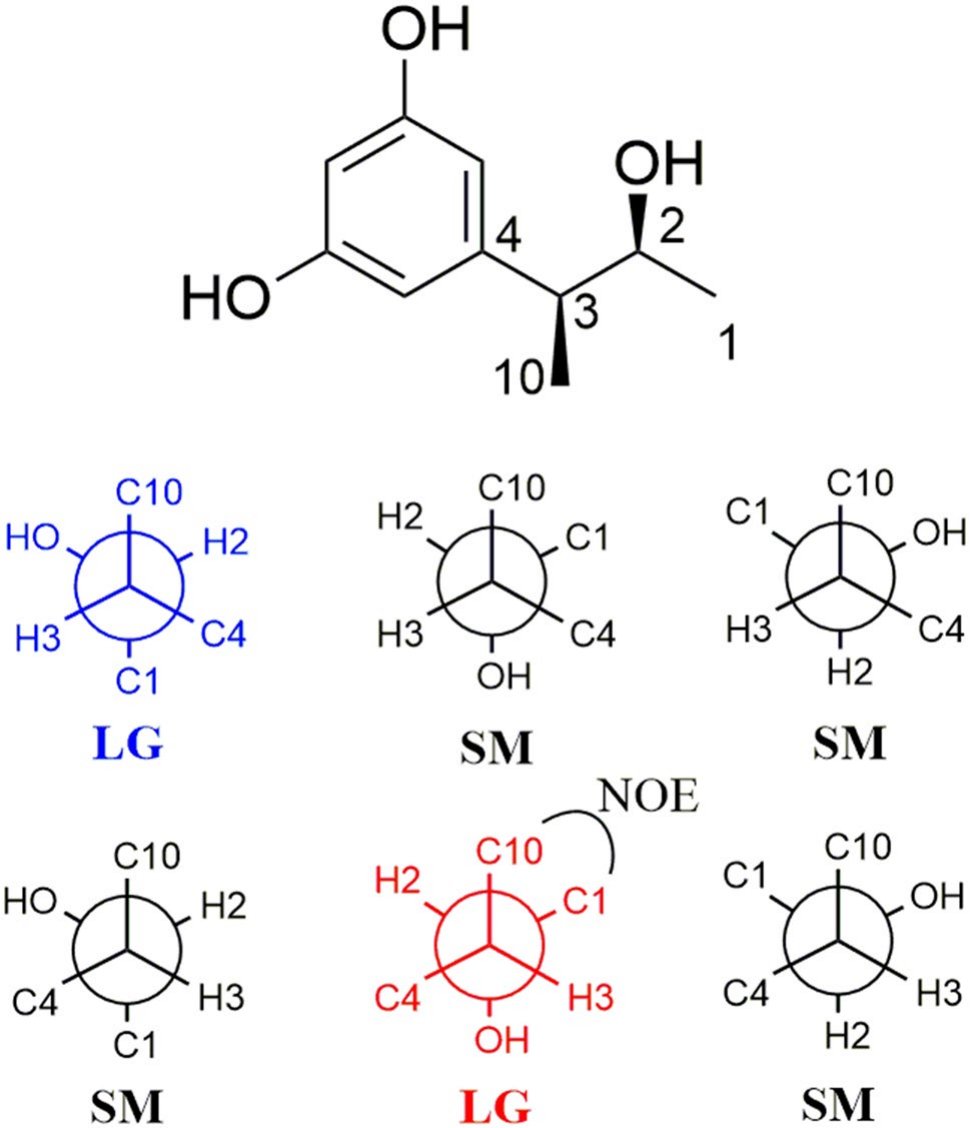
Newman projection for C-2 and C-3 of compounds **1** and **2**. Six possible relative conformations are shown: (top) 2S*,3S* and (bottom) 2R*,3S* (*LG* large coupling constant, *SM* small coupling constant)

**Fig. 4:**
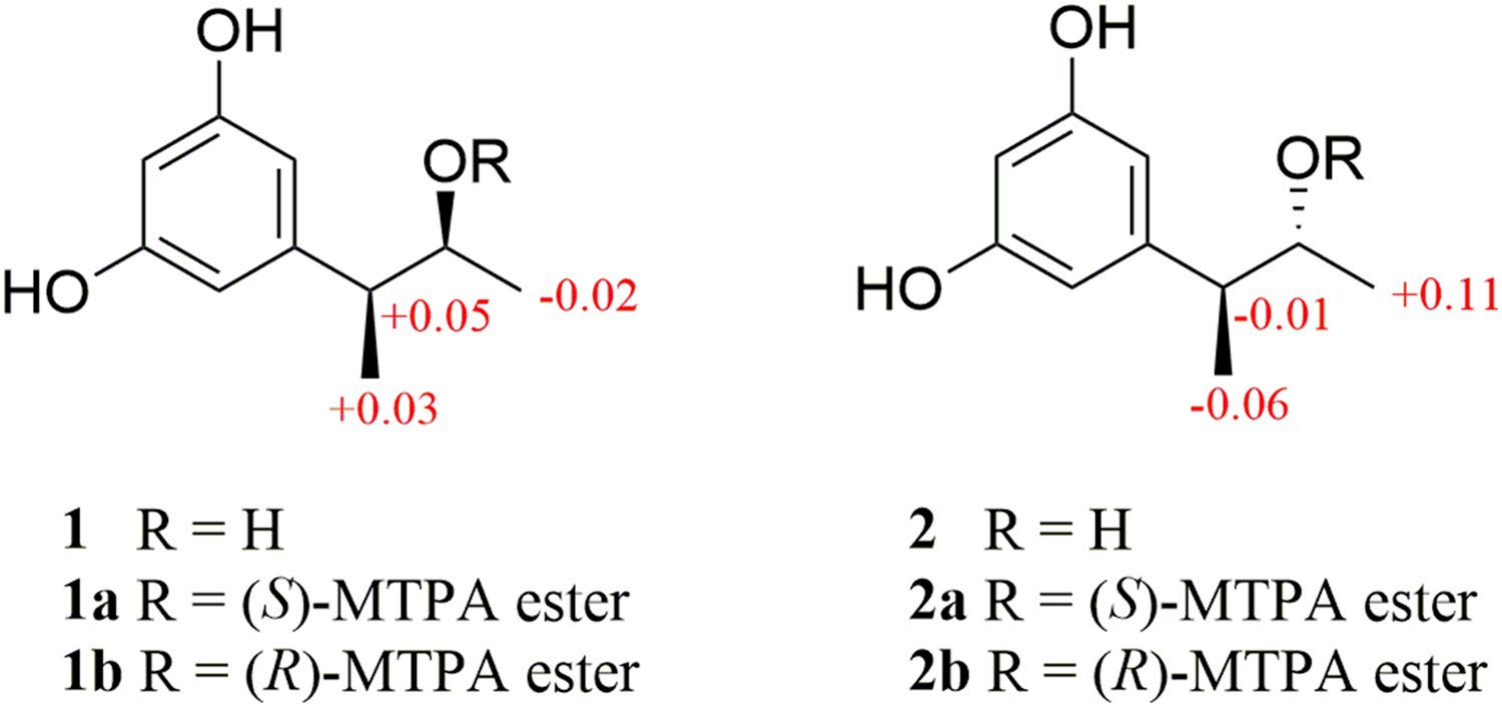
∆*δ* = *δ*_S_ − *δ*_R_ values in ppm obtained from the MTPA esters of **1** and **2**

The relative configuration of C-2 and C-3 in roussoelin A was established through selective NOESY correlations and coupling constants. A large coupling constant (^3^*J*_H-2,H-3_ = 8.3 Hz) between protons H-2 and H-3 was observed, indicating they should be in an *anti* conformation (Chlipala et al. 2010; Matsumori et al. 1999). In the analysis of *anti* conformation of roussoelin A, only two of the six possible relative conformations (blue and red color) for C-2 and C-3 were satisfied with the coupling constant (Fig. 3). A 1D selective gradient NOESY experiment revealed that H_3_-1 and H_3_-10 do not have an NOE correlation (Supplementary Figs. S8, S9), indicating a relative configuration of 2S*,3S*. The absolute configuration of the secondary alcohol was resolved by a modified version of Mosher’s method. The (*R*) and (*S*)-MTPA chloride reacted with **1**, respectively, and esterification occurred at the C-2 hydroxy group to produce the corresponding (*S*)-MTPA ester (**1a**) and (*R*)-MTPA ester (**1b**). The chemical shifts for H-1, H-3, and H-10 of **1a** and **1b** were measured as *δ*_H_ 1.18, 3.09, and 1.27 for **1a** and *δ*_H_ 1.20, 3.04, and 1.24 for **1b**, respectively. The observed differences of chemical shifts (∆*δ* = *δ*_S_ − *δ*_R_) (Fig. 4) indicated that the C-2 absolute configuration is *S*. Hence, compound **1** was identified as shown in Fig. 1 and named as roussoelin A.

Roussoelin B (**2**) was also obtained as a colorless oil and had the same molecular formula (C_10_H_14_O_3_) as roussoelin A (**1**) established by the HR-ESIMS ions at *m*/*z* 181.08712 [M^−^H]^−^ (calcd. for C_10_H_13_O_3_, 181.08702). Compound **2** shared the same planar structure as **1,** and was further identified by 2D NMR spectra (^1^H-^1^H COSY, HSQC, and HMBC) (Fig. 2). The chemical shift variation of C-1 (*δ*_C_ 22.0, *δ*_H_ 1.00 for **1**; *δ*_C_ 20.1, *δ*_H_ 1.10 for **2**), C-2 (*δ*_C_ 73.4, *δ*_H_ 3.69 for **1**; *δ*_C_ 72.8, *δ*_H_ 3.82 for **2**), C-3 (*δ*_C_ 49.6, *δ*_H_ 2.39 for **1**; *δ*_C_ 48.4, *δ*_H_ 2.58 for **2**), and C-10 (*δ*_C_ 18.7, *δ*_H_ 1.26 for **1**; *δ*_C_16.9, *δ*_H_ 1.18 for **2**), together with the different specific rotations ([α]$$\begin{array}{c}{20}\\ {\text{D}}\end{array}$$ −6.6 (c 0.20, MeOH) of **1**; [*α*]$$\begin{array}{c}{20}\\ {\text{D}}\end{array}$$ +18.5 (c 0.20, MeOH) of **2**) suggested that **2** was a stereoisomer of **1**. Similarly, the protons H-2 and H-3 were in an *anti* conformation on the base of a relative large coupling constant (^3^*J*_H-2, H-3_ = 6.3 Hz). Only two of the six possible relative conformations for C-2 and C-3 were satisfied (Fig. 3). A selective NOE experiment revealed that H_3_-1 and H_3_-10 have a strong NOE correlation (Supplementary Figs. S17, S18), indicating a relative configuration of 2R*,3S*. The stereostructure of C-2, bearing a secondary hydroxy group, was identified as *R* on the base of the modified Mosher’s method compared to the chemical shifts for H-1, H-3, and H-10 (**1a** *δ*_H_ 1.19, 3.02, and 1.22; **1b** *δ*_H_ 1.08, 3.03, and 1.28) (Fig. 4). Thus, roussoelin B (**2**) was 2-epimer of roussoelin A.

The known compounds, 4-hydroxyscytalone (**3**) (Cimmino et al. 2016), 4,6,8-trihydroxy-3,4-dihydronaphthalen-1(2*H*)-one (6-hydroxyisosclerone) (**4**) (Yan et al. 2008), acremonone F (**5**) (Angelie et al. 2002), xestodecalactone A (**6**) (Angelie et al. 2002), corynechromone K (**7**) (Dong-Lin et al. 2015), corynechromone A (**8**) (Dong-Lin et al. 2015), (3*Z*,5*S*,6*E*,8*S*,9*S*,10*R*)-8-chloro-5,8,9,10-tetrahydro-5,9-dihydroxy10-methyl-2*H*-oxecin-2-one (**9**) (Greve et al. 2008; Zheng et al. 2015), modiolide A (**10**) (Greve et al. 2008), curvulide B1 (**11**) (Greve et al. 2008), and curvulide B2 (**12**) (Greve et al. 2008) were verified by ^1^H and ^13^C NMR, ESI–MS, and optical rotation data analysis, as well as comparison of spectroscopic data with literature.

**Fig. 5:**
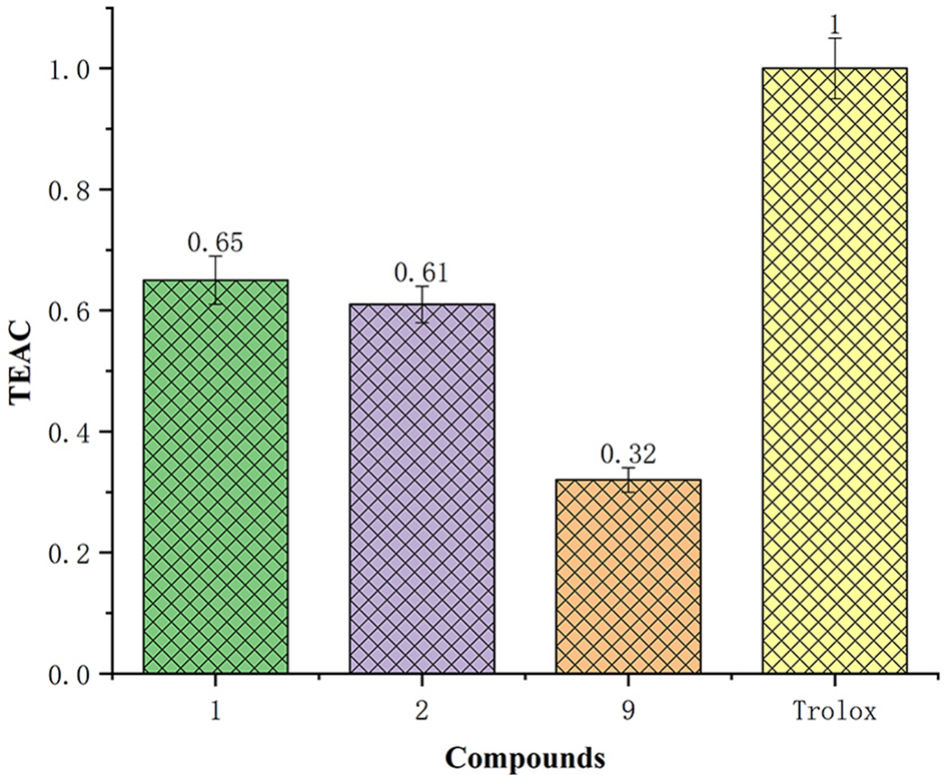
Antioxidant capacity of compounds **1**, **2**, and **9** as determined by ABTS

All isolated compounds were tested for their anti-inflammatory activity in vitro by inhibition of LPS-activated NO production in RAW264.7 cells with the Griess assay and their cytotoxicity using MCF-7 (breast cancer), HepG2 (liver cancer), and A549 (lung cancer) human cell lines. None of them showed inhibition activity or cytotoxicity at 50 μmol/L. Compounds **1**–**12** were also evaluated using the total antioxidant capacity assay kit with a rapid ABTS method. Only compounds **1**, **2**, and **9** showed moderate total antioxidant capacity (0.65 of **1**; 0.61 of **2**; 0.32 of **9**) with Trolox as a positive control (Fig. 5). Phenolic compounds (including cinnamic acids, benzoic acids, flavonoids, proanthocyanidins, coumarins, stilbenes, lignans, and lignins) are the most widespread class of metabolites in nature (Pereira et al. 2009). The antioxidant capacity of phenolic compounds **1** and **2** should be attributed to their ability to chelate metal ions involved in the production of free radicals and suggests that chemical protection of symbiotic microbes are benefitial to ascidians screening UV or inhibiting enzymes involved in radical generation (Cos et al. 1998).

## Materials and methods

### General experimental procedures

Optical rotations were measured on an MCP 200 polarimeter (Anton Paar, China). Infrared spectroscopy was performed on a Fourier transformation infrared spectrometer coupled with infrared microscope EQUINOX 55 (Bruker, Germany). 1D and 2D NMR data were measured on Bruker Avance 400 or 600 MHz spectrometers (Bruker, Germany) using tetramethylsilane (TMS) as the internal standard. Electrospray mass spectrometry (ESIMS) was obtained on an ACQUITY QDA (Waters Corporation, USA). High resolution electrospray mass spectrometry (HR-ESIMS) was tested on an LTQ-Orbitrap LC–MS spectrometer (Thermo Corporation, USA). Column chromatography was carried out on silica gel with 200–300 mesh (Qingdao Marine Chemical Factory, China) and Sephadex LH-20 (GE Healthcare, UK). High performance liquid chromatography (HPLC) was performed on on Essentia LC-16 with an SPD-16 Detector (Shimadzu, China).

### Fungal material

In this study, the fungus SYSU-MS4723 was isolated from an ascidian *Styela plicata*, which was collected in the Mirs Bay (22°33′22.1′′N, 114°27′09.3′′E), Shenzhen, Guangdong Province, China, in April 2016. Purified fungus was isolated from ascidian on the base of the standard protocol (Kjer et al. 2010). The strain was identified to be *R. siamensis* SYSU-MS4723 on the base of morphological characteristics and the ITS region (Raja et al. 2017). The sequence data of the fungal strain have been submitted and deposited at GenBank with accession no. MH465397. The voucher specimen was preserved on potato dextrose agar slants at 4 °C at the School of Marine Sciences, Sun Yat-Sen University.

### Extraction and isolation

The strain SYSU-MS4723 was cultured in autoclaved solid-substrate rice medium on sixty Erlenmeyer flasks (each flask containing 60 ml rice and 60 ml 3% artificial sea water) for 30 days under static conditions and daylight. Following incubation, the fungal solid-substrate rice medium was extracted three times with MeOH solvent to afford the crude extract. The crude extract was then extracted three times with EtOAc solvent and evaporated under reduced pressure to give a dark brown residue (18.5 g). The EtOAc extract residue was then subjected to flash column chromatography on silica gel eluted by a gradient of petroleum ether/EtOAc from 100:0 to 0:100 to separate into seven fractions (Fr. A–Fr. G). Fraction B was divided into five subfractions Fr.B.1–Fr.B.5 by Sephadex LH-20 (CC, 3 × 50 cm) eluting with MeOH-CH_2_Cl_2_ (*v*/*v*, 1:1). Fr.B.3 was subsequently performed on silica gel CC eluted by PE-EtOAc (*v*/*v*, 70:30) to give Fr.B.3.1–Fr.B.3.6. Then compound **6** (3 mg) was purified from Fr.B.3.3 subjected to Sephadex LH-20 (CC, 3 × 50 cm) and eluted with MeOH-CH_2_Cl_2_ (*v*/*v*, 1:1). Fr.B.3.4 was purified by the semi-preparative PR-HPLC (MeOH-H_2_O, *v*/*v*, 75:25, 1.5 ml/min, ultimate C_18_ column 10 × 250 nm, 5 μm) to yield compound **7** (3 mg, *t*_R_ = 15.5 min). Compound **8** (3 mg) was directly purified from Fr.B.4 performed on silica gel CC by elution with PE-EtOAc (*v*/*v*, 70:30), while compounds **3** (4 mg) and **4** (5 mg) were isolated from Fr.B.3.5 using the silica gel CC eluted by MeOH-CH_2_Cl_2_ (*v*/*v*, 3:97). Then Fr. C was subjected to Sephadex LH-20 (MeOH-CH_2_Cl_2_, *v*/*v*, 1:1) to produce Fr.C.1–Fr.C.6, and Fr.C.4 was chromatographed on a silica gel with MeOH-CH_2_Cl_2_ (4:96) to afford five subfractions (Fr.C.4.1–Fr.C.4.5). The new compounds **1** (4 mg, *t*_R_ = 17 min) and **2** (4 mg, *t*_R_ = 18 min) were purified by semi-preparative PR-HPLC (MeOH-H_2_O, *v*/*v*, 75:25, 1.5 ml/min, ACE 5 C18-PFP column 250 × 10 mm, 5 μm) from Fr.C.4.4. The fourth fraction D was applied to a Sephadex LH-20 (MeOH-CH_2_Cl_2_, *v*/*v*, 1:1) to yield Fr.D.1–Fr.D.5. Subsequently, compounds **11** and **12** (3 mg, *t*_R_ = 23.5 min; 2 mg, *t*_R_ = 24.3 min) were purified from Fr.D.5 by semi-preparative PR-HPLC (MeOH-H_2_O, *v*/*v*, 70:30, 1.5 ml/min, ACE 5C18-AR column 250 × 10 mm, 5 μm). Fr. E was also applied to Sephadex LH-20 (MeOH-CH_2_Cl_2_, *v*/*v*, 1:1) to yield Fr.E.1–Fr.E.5. Fr.E.4 was chromatographed on a silica gel column with PE-EtOAc (*v*/*v*, 50:50) to give four subfractions (Fr.E.4.1–Fr.E.4.5). Fr.E.4.3 was performed on silica gel CC eluted by MeOH-CH_2_Cl_2_ (*v*/*v*, 5:95) to afford **5** (3 mg) and **9** (6 mg). And Fr.E.4.5 was subject to silica gel CC eluted by MeOH-CH_2_Cl_2_ (*v*/*v*, 5:95) to obtained **10** (4 mg).

**Roussoelin A (1)**: colorless oil; [*α*]$$\begin{array}{c}{20}\\ {\text{D}}\end{array}$$ −6.6 (*c* 0.20, MeOH); IR (neat) *v*_max_ 3346, 2978, 2918, 2850, 1601, 1462, 1329, 1151, 1084, 989, 931, 839, 700 cm^−1^; ^1^H NMR (400 MHz, CD_3_OD) and ^13^C NMR (100 MHz, CD_3_OD) data see Table 1; HR-ESIMS *m/z* 181.08712 [M^−^H]^−^ (calcd. for C_10_H_13_O_3_, 181.08702).

**Roussoelin B (2)**: colorless oil; [α]$$\begin{array}{c}{20}\\ {\text{D}}\end{array}$$ 18.5 (*c* 0.20, MeOH); IR (neat) *v*_max_ cm^−1^ 3329, 2972, 2924, 1603, 1454, 1342, 1149, 997, 841, 700; ^1^H NMR (400 MHz, CD_3_OD) and ^13^C NMR (100 MHz, CD_3_OD) data see Table 1; HR-ESIMS *m*/*z* 181.08712 [M^−^H]^−^ (calcd. for C_10_H_13_O_3_, 181.08702).

### Preparation of (*S*)-MTPA ester and (*R*)-MTPA ester

#### (*S*)-MTPA ester (1a) and (*R*)-MTPA ester (1b)

Compound **1** (1.0 mg) dissolved in pyridine-*d*_5_ (0.5 ml) in an NMR tube, and then (*R*)-MPTACl (5.0 μl) was added to react at room temperature for 24 h. Then the ^1^H NMR spectrum of the (*S*)-MTPA ester derivative (**1a**) was measured directly on the reaction mixture (Hoye et al. 2007; Zhang et al. 2017). ^1^H NMR (selected signals, pyridine-*d*_5_, 400 MHz) *δ*_H_: 1.18 (3H, *d*, H-1), 3.09 (1H, *m*, H-3), 1.27 (3H, *d*, H-10).

Similarly, another reaction of **1** (1.0 mg), (*S*)-MPTACl (5.0 μl), and pyridine-*d*_5_ (0.5 ml) was performed as described above for **1a** to afford **1b**. ^1^H NMR (selected signals, pyridine-*d*_5_, 400 MHz) *δ*_H_: 1.20 (3H, *d*, H-1), 3.04 (1H, *m*, H-3), 1.24 (3H, *d*, H-10).

#### (*S*)-MTPA ester (2a) and (*R*)-MTPA ester (2b)

(*S*)-MTPA ester (**2a**) and (*R*)-MTPA ester (**2b**) were obtained by refering to the above method. ^1^H NMR (selected signals, pyridine-*d*_5_, 400 MHz) **2a** *δ*_H_: 1.19 (3H, *d*, H-1), 3.02 (1H, *m*, H-3), 1.22 (3H, *d*, H-10). **2b** *δ*_H_: 1.08 (3H, *d*, H-1), 3.03 (1H, *m*, H-3), 1.28 (3H, *d*, H-10).

### Cytotoxic assay

All compounds were tested for cytotoxicity against MCF-7 (breast cancer), HepG2 (liver cancer), and A549 (lung cancer) human cancer cell lines. Human cancer cell lines were purchased from the cell bank of the Chinese Academy of Sciences (Shanghai, China). The cytotoxicity assay was based on the MTT method according to previously reported procedures (Chen et al. 2016).

### Anti-inflammatory assay

All compounds were tested for their anti-inflammatory activity on the basis of previously reported procedures (Zhang et al. 2019).

### Total antioxidant capacity assay

Total antioxidant capacity assay kit with a rapid ABTS method (Beyotime Institute of Biotechnology, China) was used to evaluate the total antioxidant capacity based on the manufacturer’s instructions. Samples were incubated at 25 °C for 6 min and then were recorded at 414 nm using a multimode reader (Thermo Fisher Scientific, USA).

## Electronic supplementary material

Below is the link to the electronic supplementary material.

Supplementary file1 (DOCX 1636 kb)
