# Supplementary material for: Roussoelins A and B: two phenols with antioxidant capacity from ascidian-derived fungus Roussoella siamensis SYSU-MS4723
Source: Mar Life Sci Technol. 2020 Oct 23;3(1):69–76. doi: 10.1007/s42995-020-00066-8 (PMC10064353; doi:10.1007/s42995-020-00066-8)

**Supplementary Figures**

Roussoelins A and B: two phenols with antioxidant capacity from ascidian-derivedfungus *Roussoella siamensis* SYSU-MS4723

Senhua Chen ^1,3^, Hongjie Shen ^1^, Yanlian Deng ^2^, Heng Guo ^1^, Minghua Jiang ^1^, Zhenger Wu ^1^, Huimin Yin ^1^, Lan Liu ^1,3,^*

^1.^ School of Marine Sciences, Sun Yat-sen University, Guangzhou 510006, China

^2.^ School of Pharmacy, Guangdong Medical University, Dongguan 523808, China

^3.^ Southern Laboratory of Ocean Science and Engineering (Guangdong, Zhuhai), Zhuhai 519000, China

***** Correspondence: cesllan@mail.sysu.edu.cn (L.L.); Tel.: +86-020-84725459.

# **Supplementary Fig. S1** HR-ESIMS of **1**


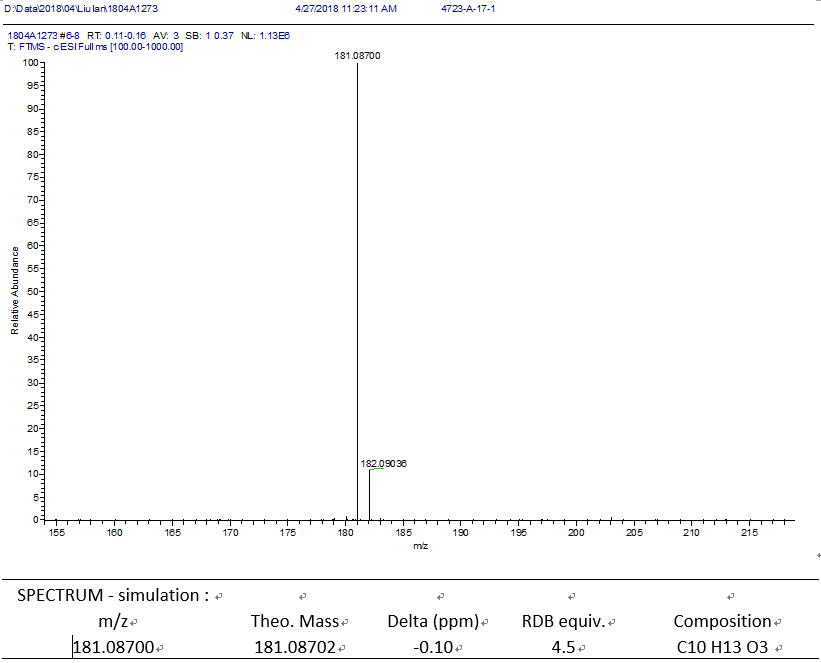


# **Supplementary Fig. S2** IR spectrum of **1**





# **Supplementary Fig. S3** ^1^H (400 MHz) NMR spectrum of **1** in CD_3_OD


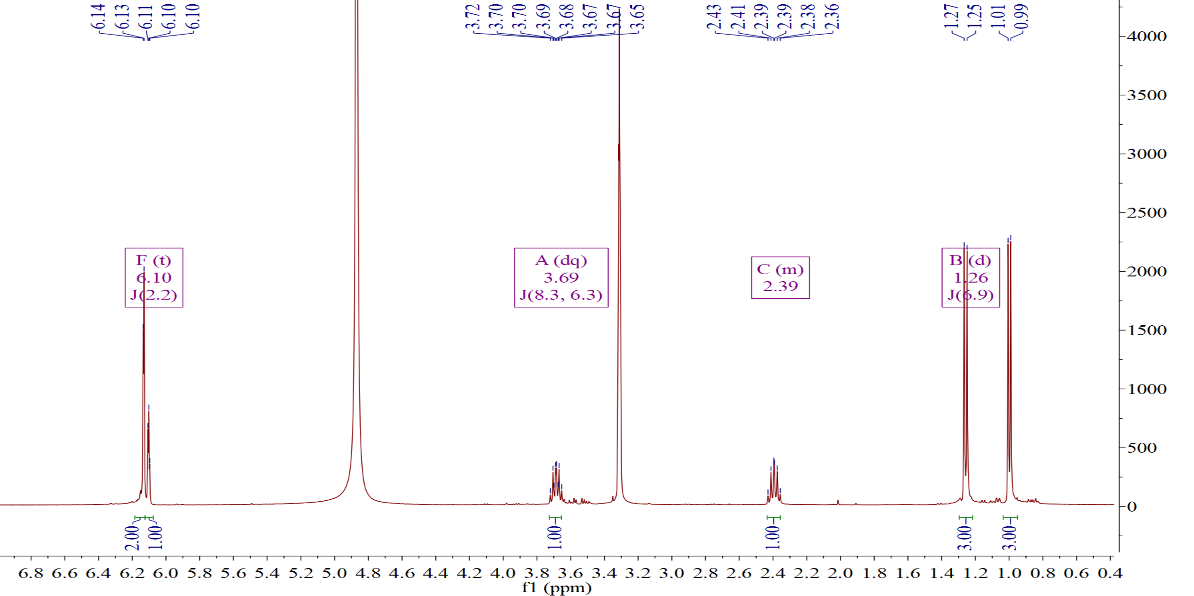


# **Supplementary Fig. S4** ^13^C NMR spectrum of **1** in CD_3_OD


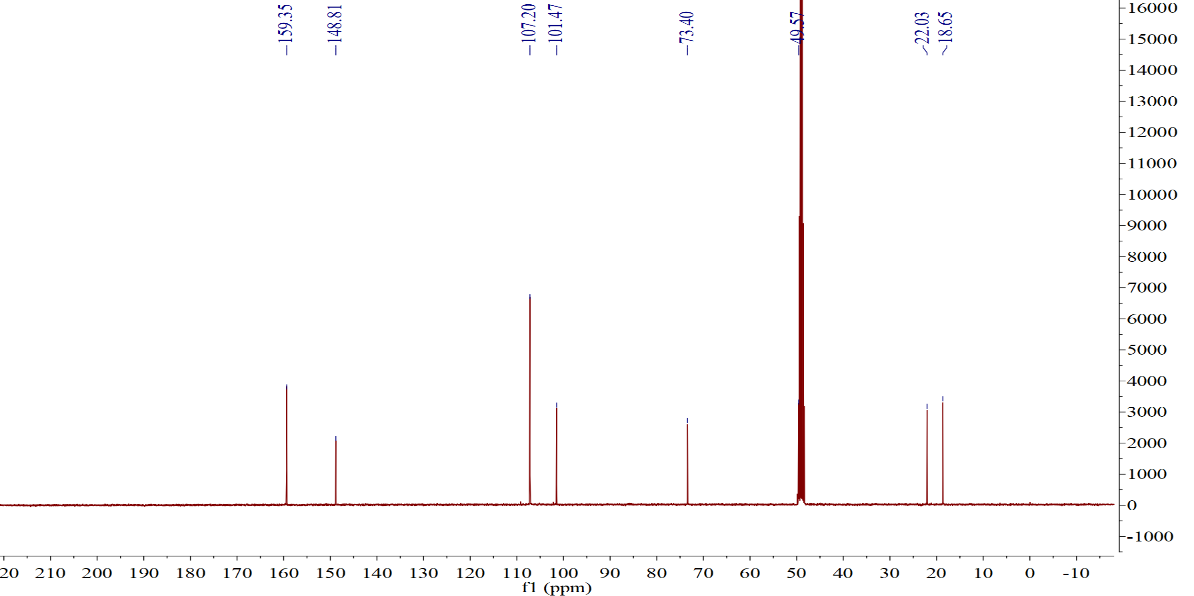


# **Supplementary**
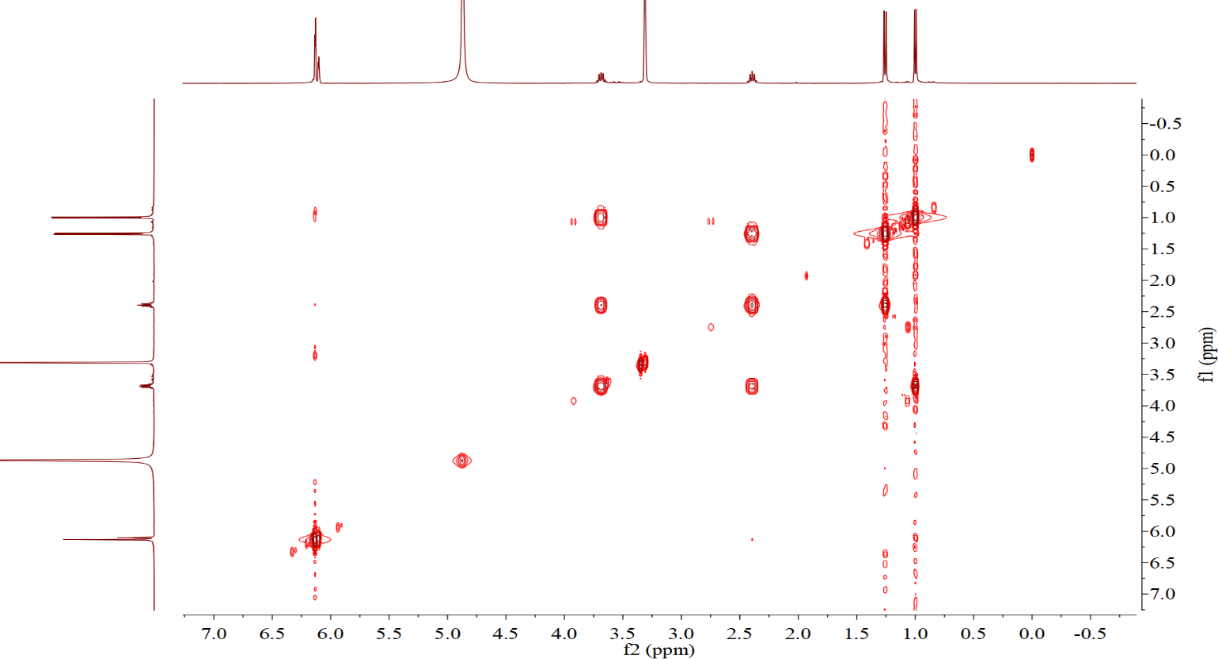
**Fig. S5** ^1^H-^1^H COSY spectrum of **1** in CD_3_OD

# **Supplementary Fig. S6** HSQC spectrum of **1** in CD_3_OD


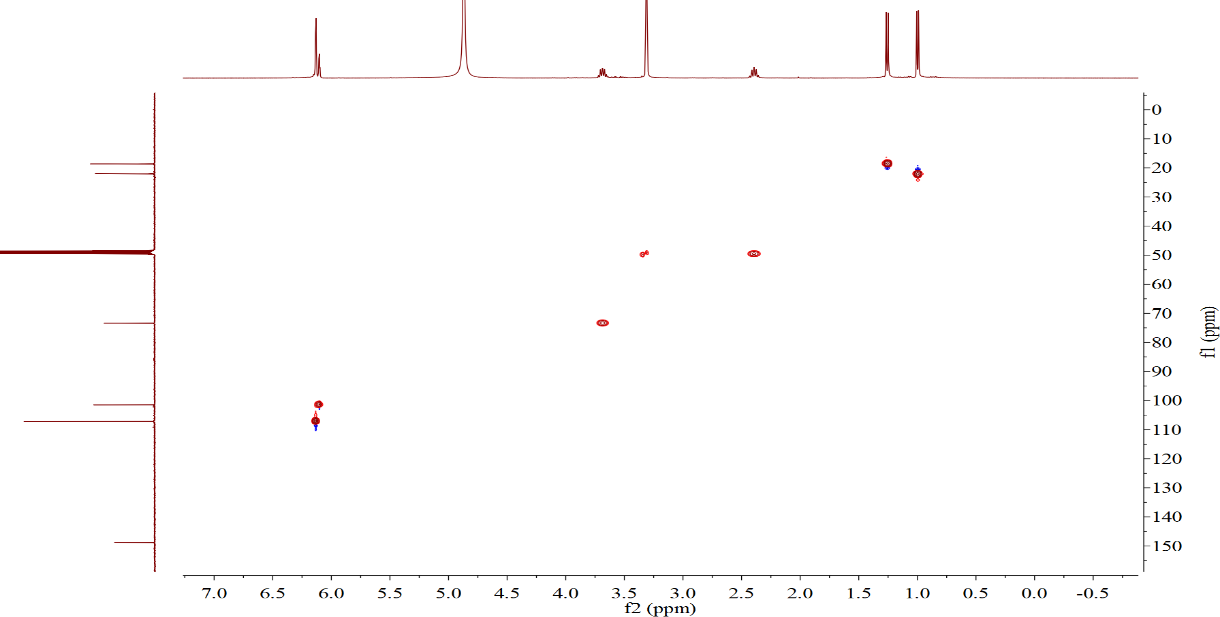


# **Supplementary Fig. S7** HMBC spectrum of **1** in CD_3_OD


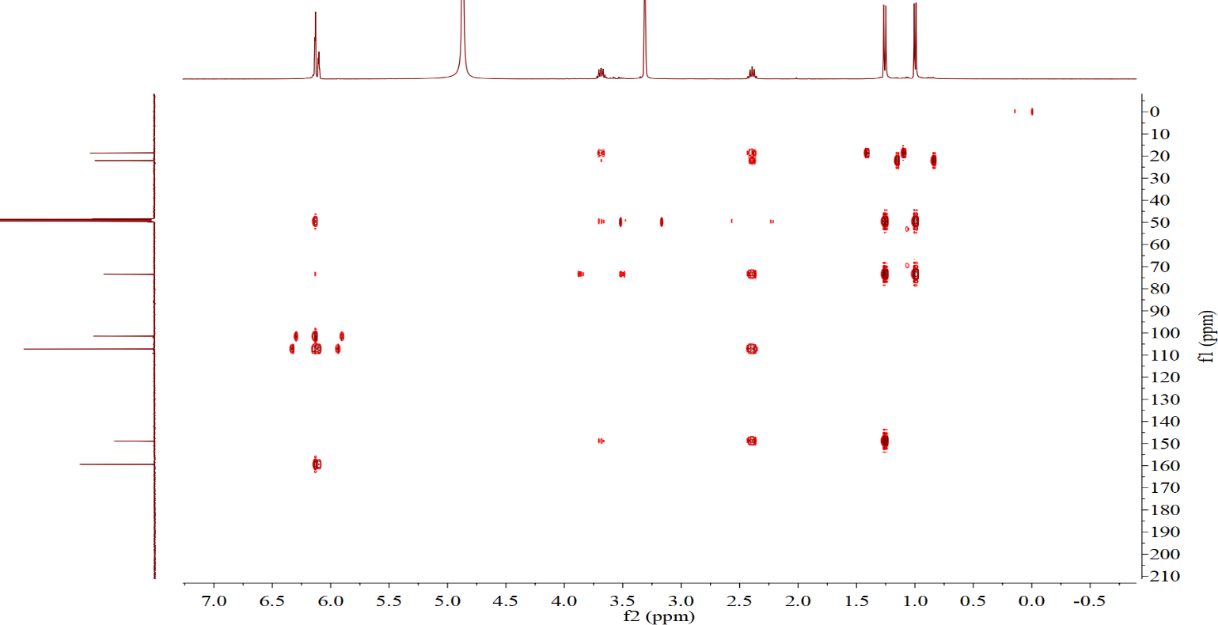


# **Supplementary Fig. S8** Selective Gradient NOESY spectrum (600 MHz) (1.276 ppm) of **1** in CD_3_OD


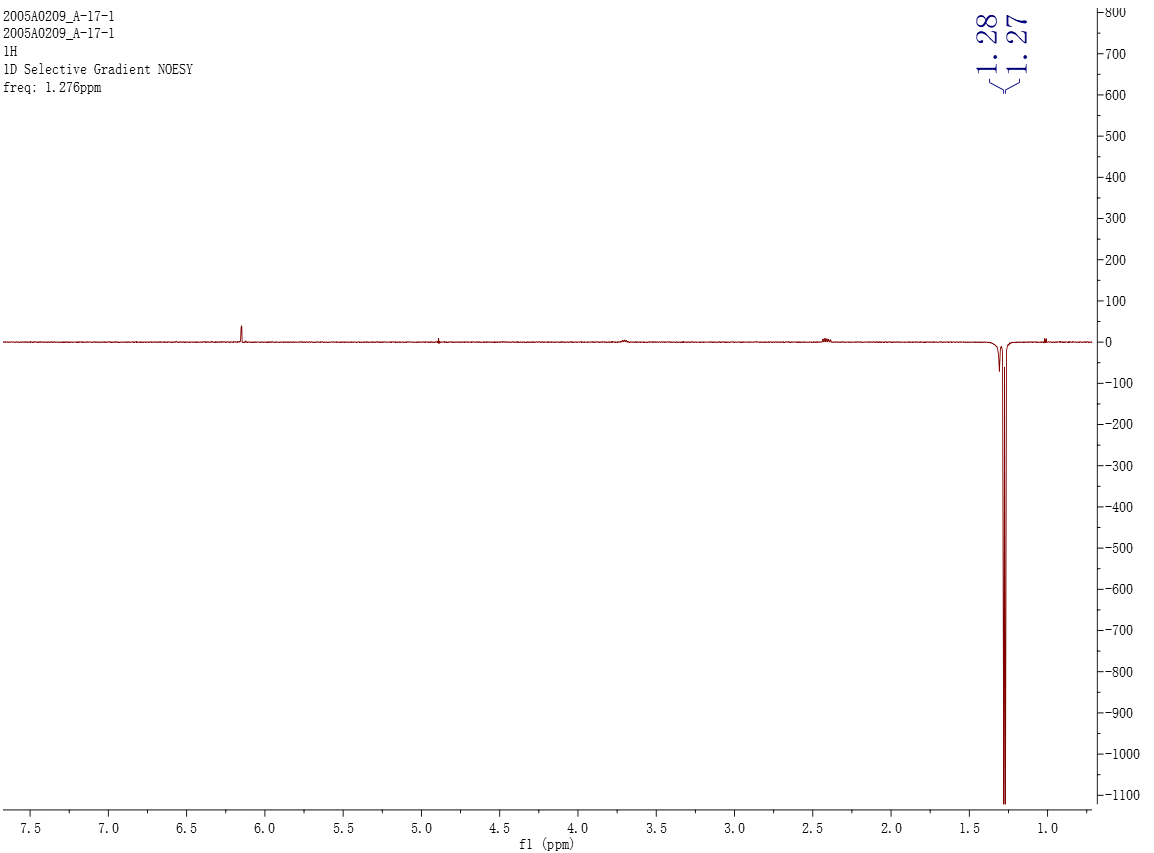


# **Supplementary Fig. S9** Selective Gradient NOESY spectrum (600 MHz) (1.015 ppm) of **1** in CD_3_OD


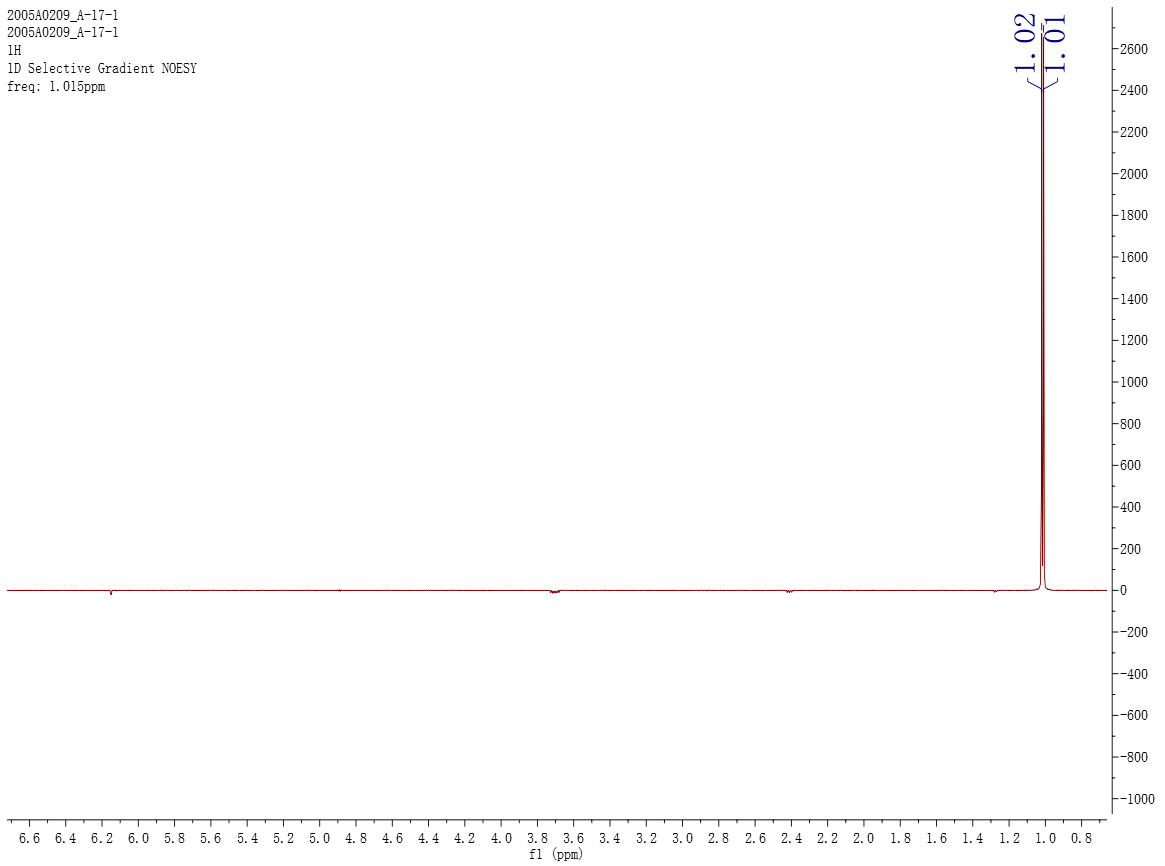


# **Supplementary Fig. S10** HR-ESIMS of **2**


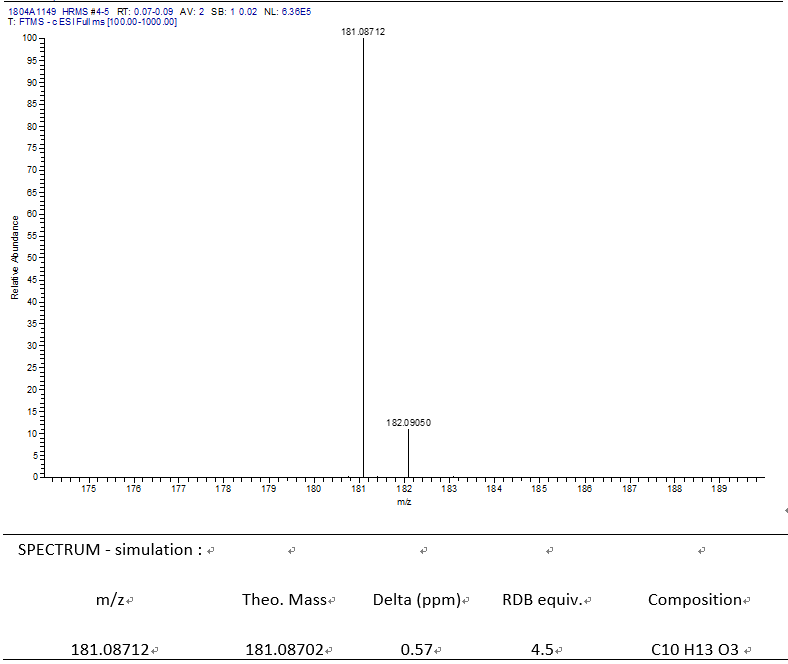


# **Supplementary Fig. S11** IR spectrum of **2.**





# **Supplementary Fig. S12** ^1^H (400 MHz) NMR spectrum of **2** in CD_3_OD


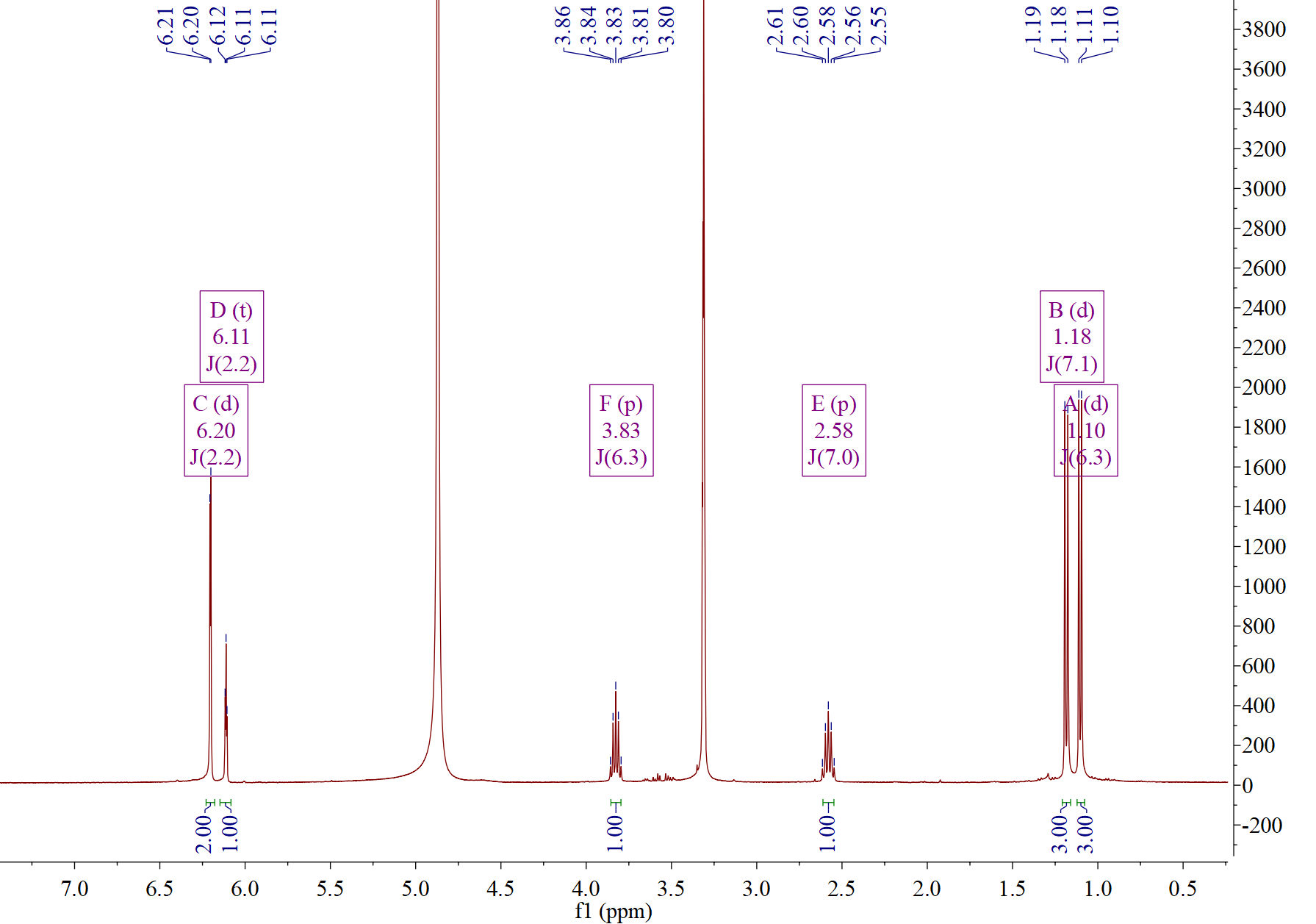


# **Supplementary Fig. S13** ^13^C NMR spectrum of **2** in CD_3_OD


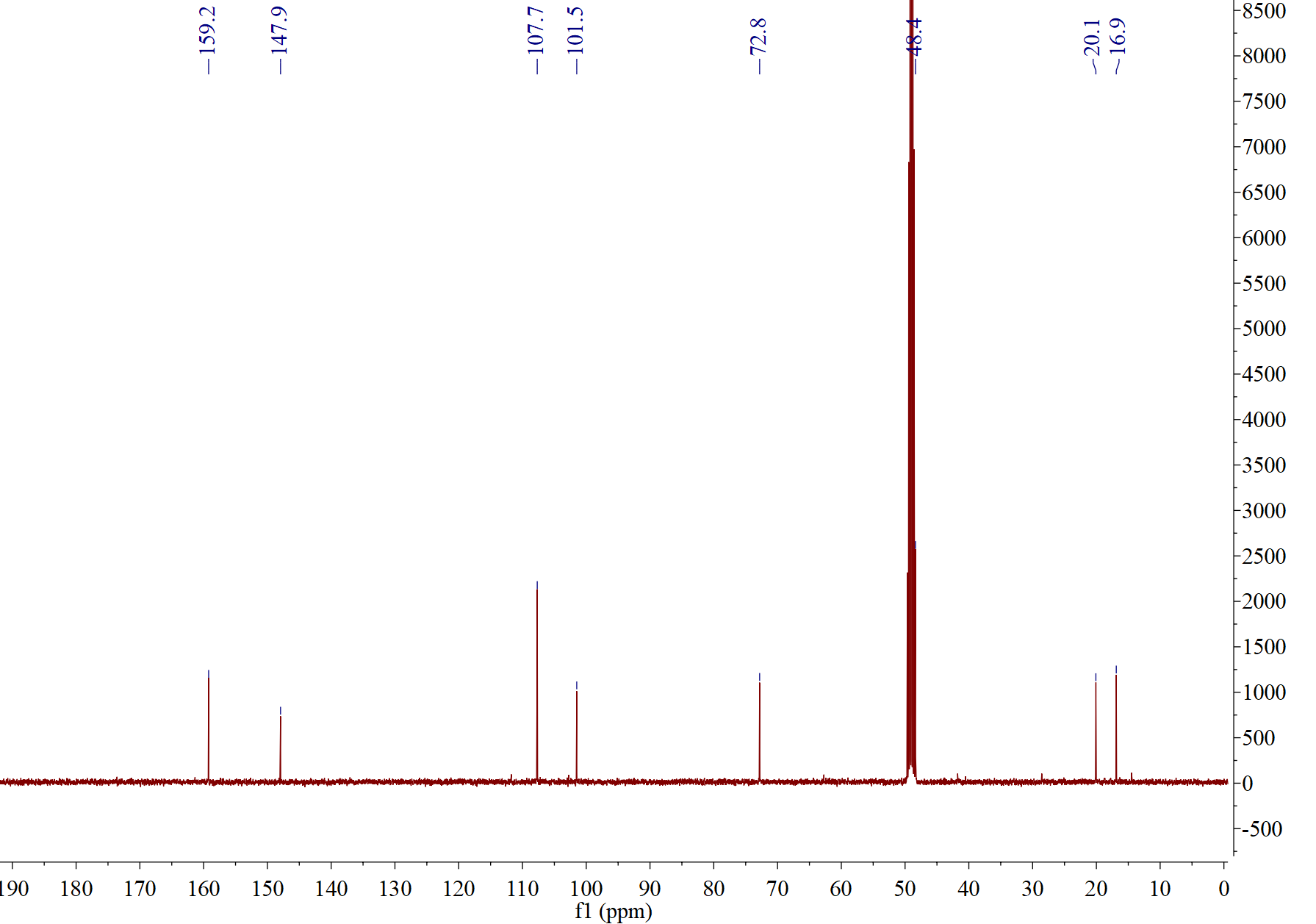


# **Supplementary Fig. S14** ^1^H-^1^H COSY spectrum of **2** in CD_3_OD


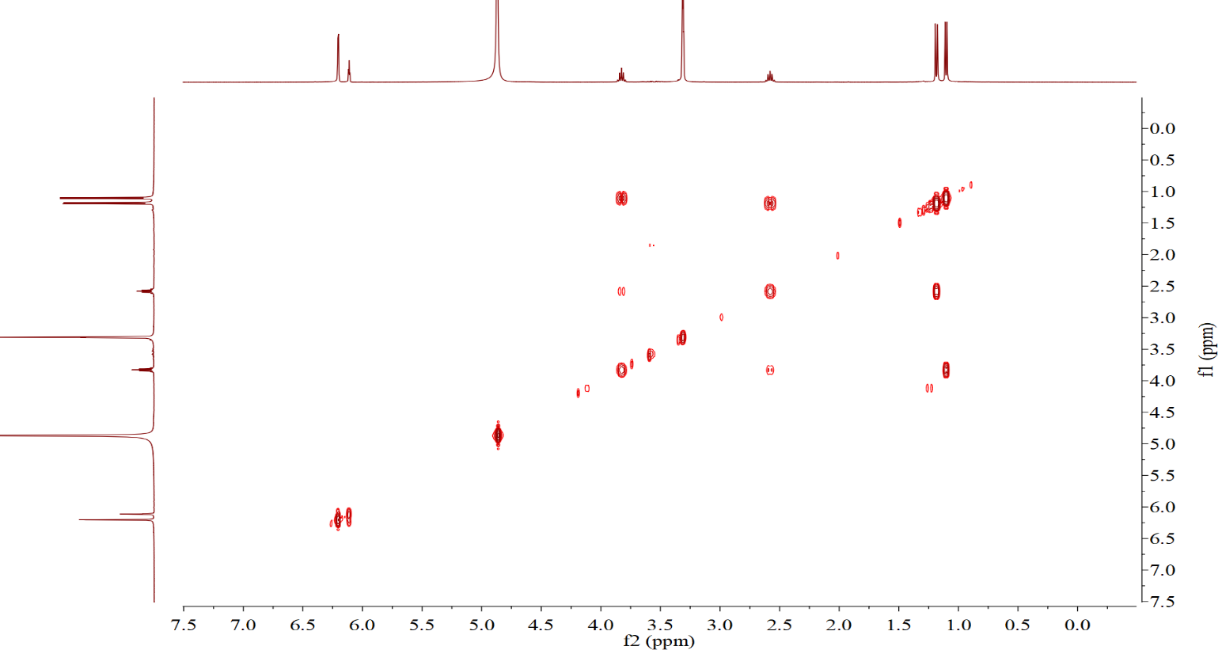


# **Supplementary Fig. S15** HSQC spectrum of **2** in CD_3_OD


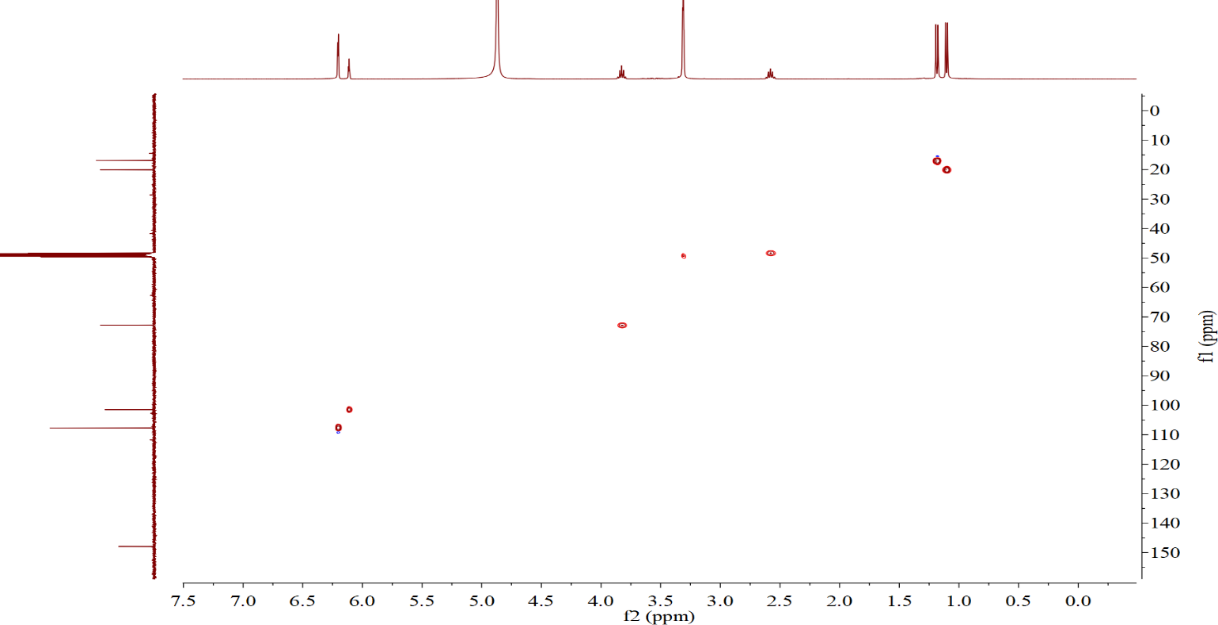


# **Supplementary Fig. S16** HMBC spectrum of **2** in CD_3_OD


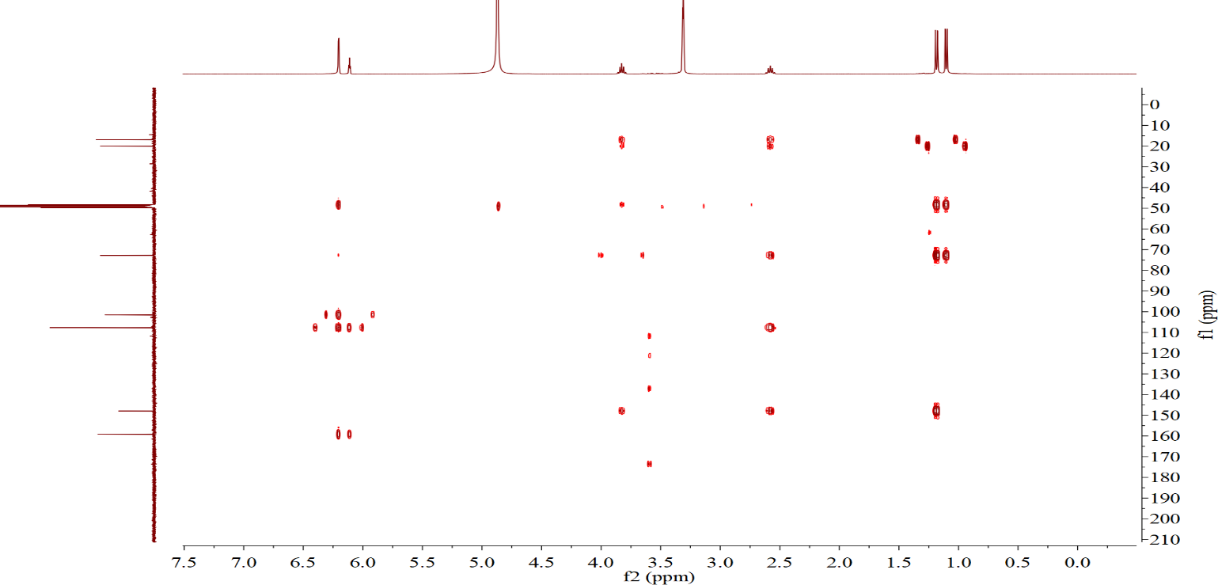


# **Supplementary Fig. S17** Selective Gradient NOESY spectrum (600 MHz) (1.122 ppm) of **1** in CD_3_OD


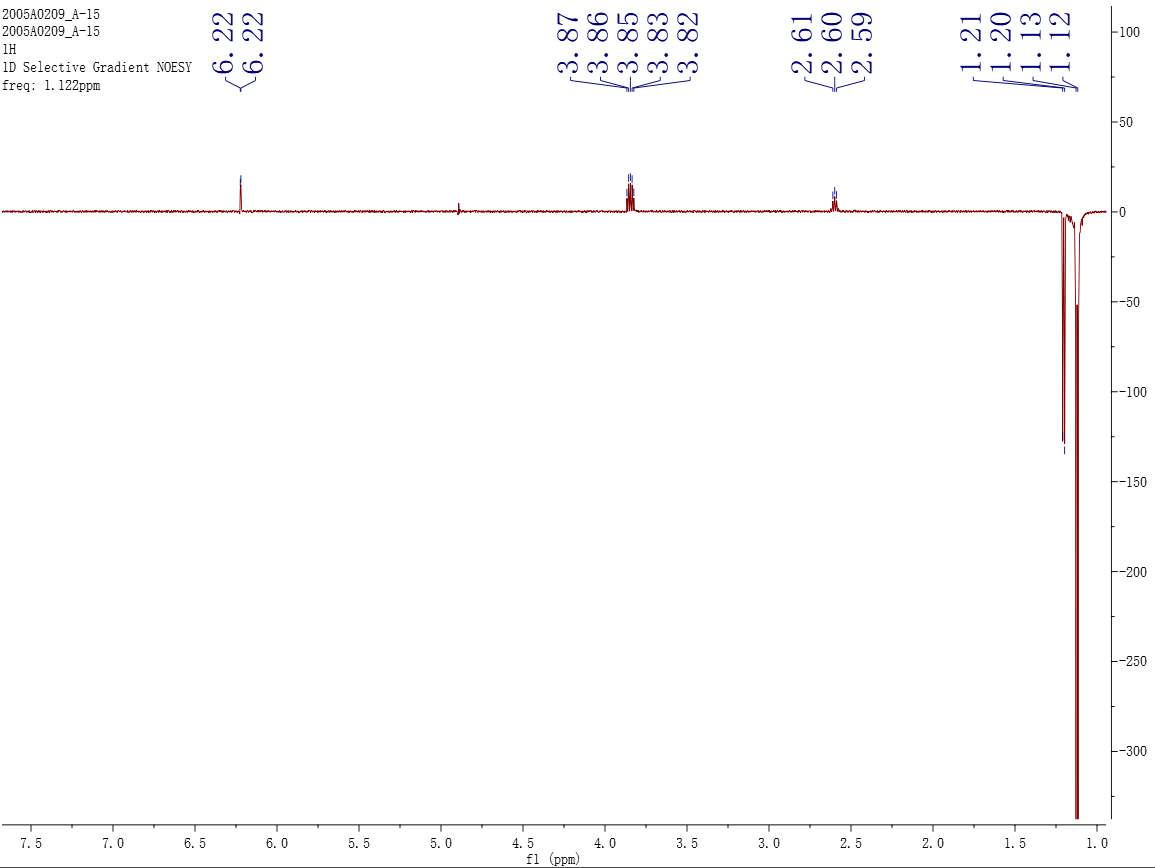


# **Supplementary Fig. S18** Selective Gradient NOESY spectrum (600 MHz) (1.204 ppm) of **1** in CD_3_OD


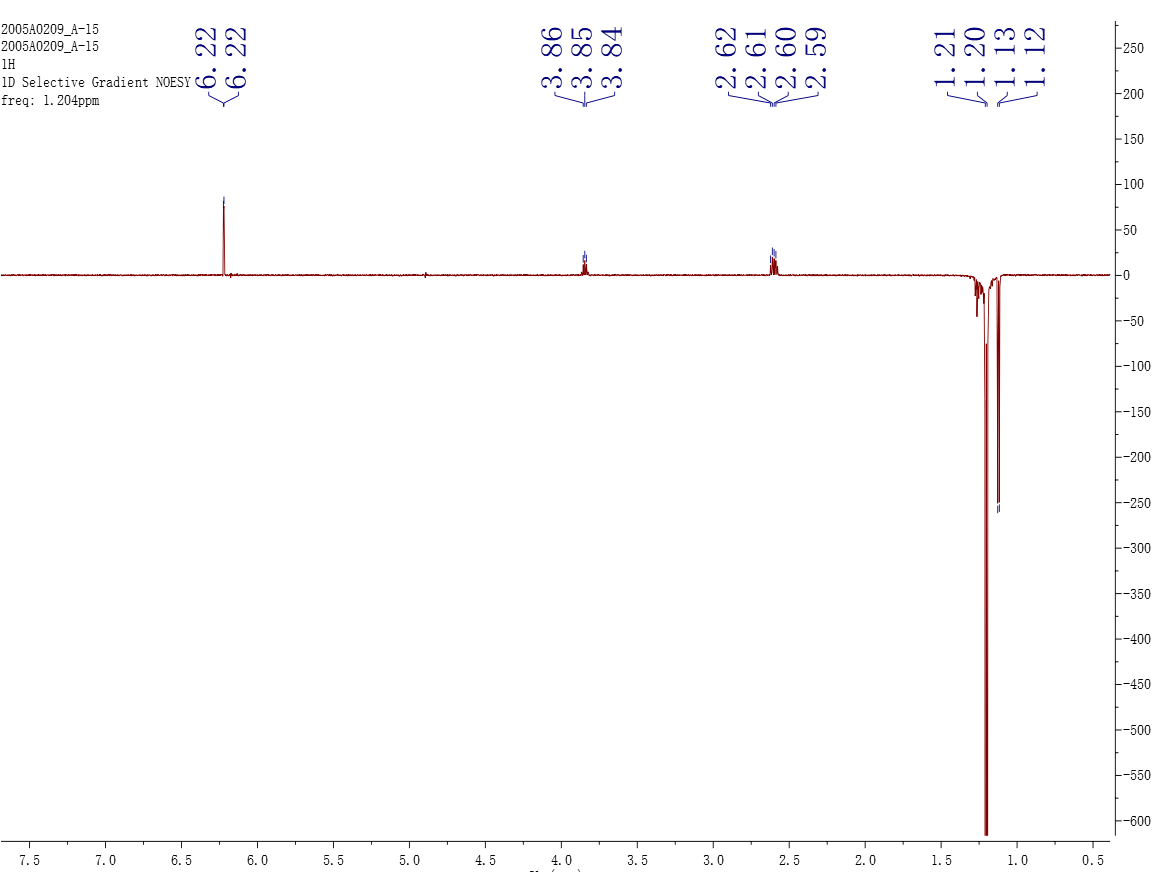


# **Supplementary Fig. S19** ^1^H (400 MHz) NMR spectrum of **1a** in pyridine-*d*_5_


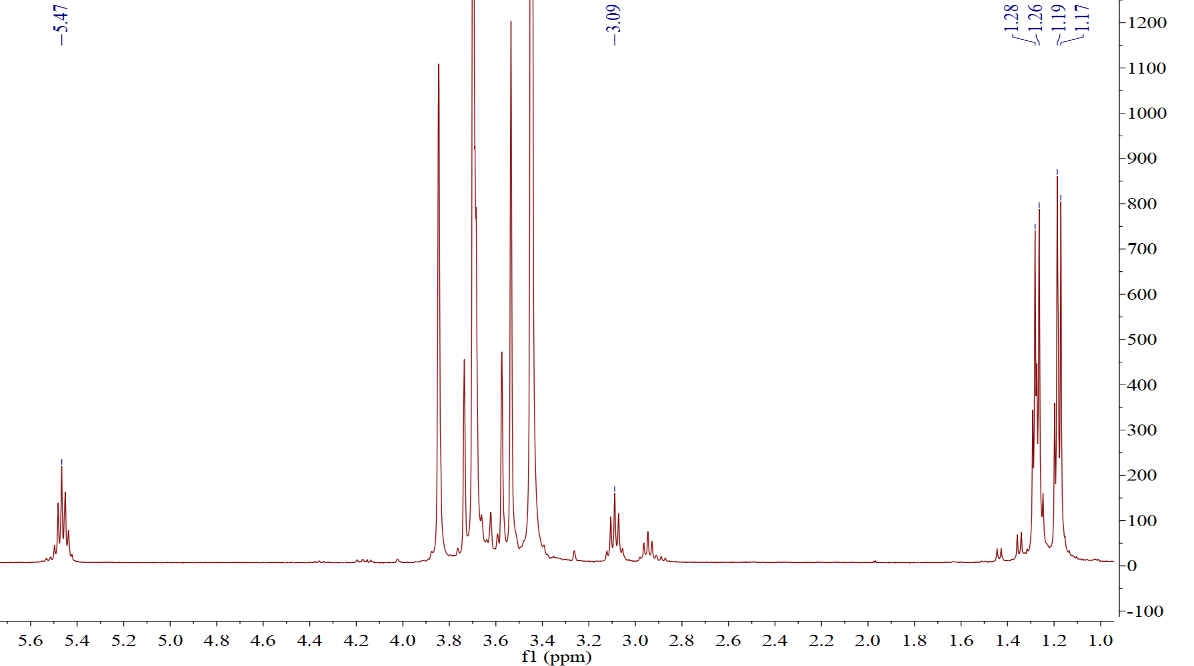


# **Supplementary Fig. S20** ^1^H (400 MHz) NMR spectrum of **1b** in pyridine-*d*_5_


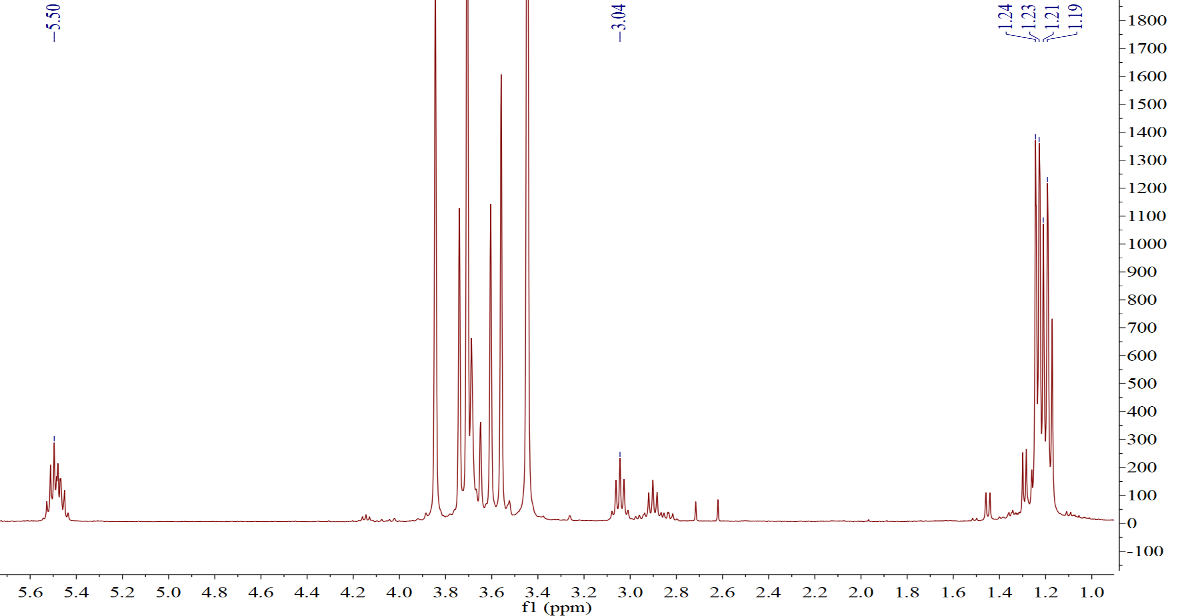


# **Supplementary Fig. S21** ^1^H (400 MHz) NMR spectrum of **2a** in pyridine-*d*_5_


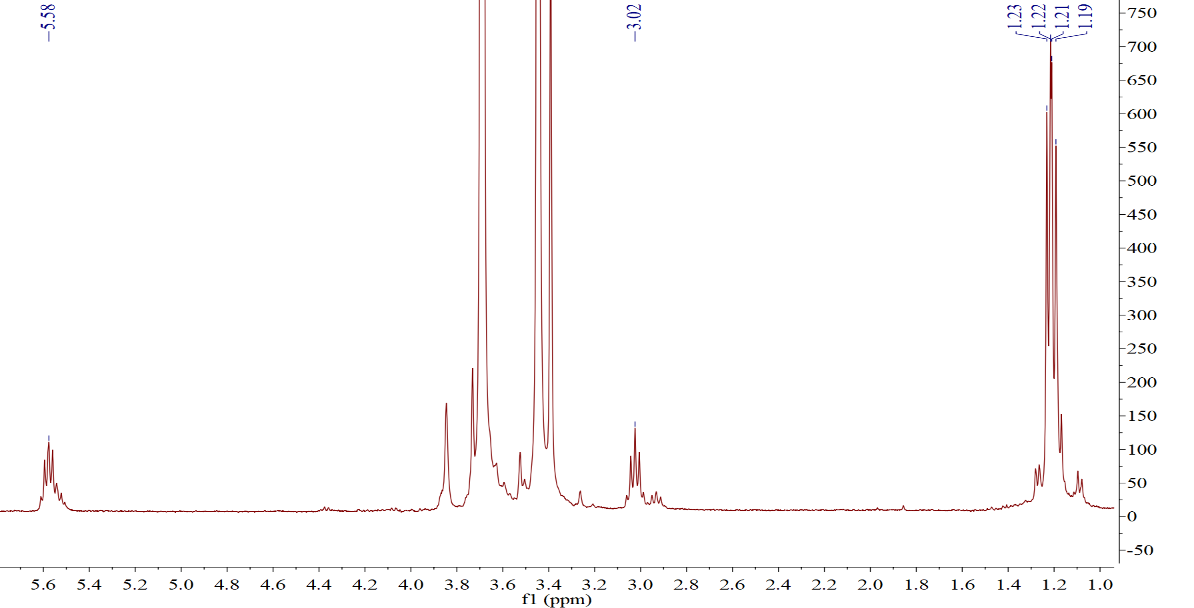


# **Supplementary Fig. S22** ^1^H (400 MHz) NMR spectrum of **1b** in pyridine-*d*_5_


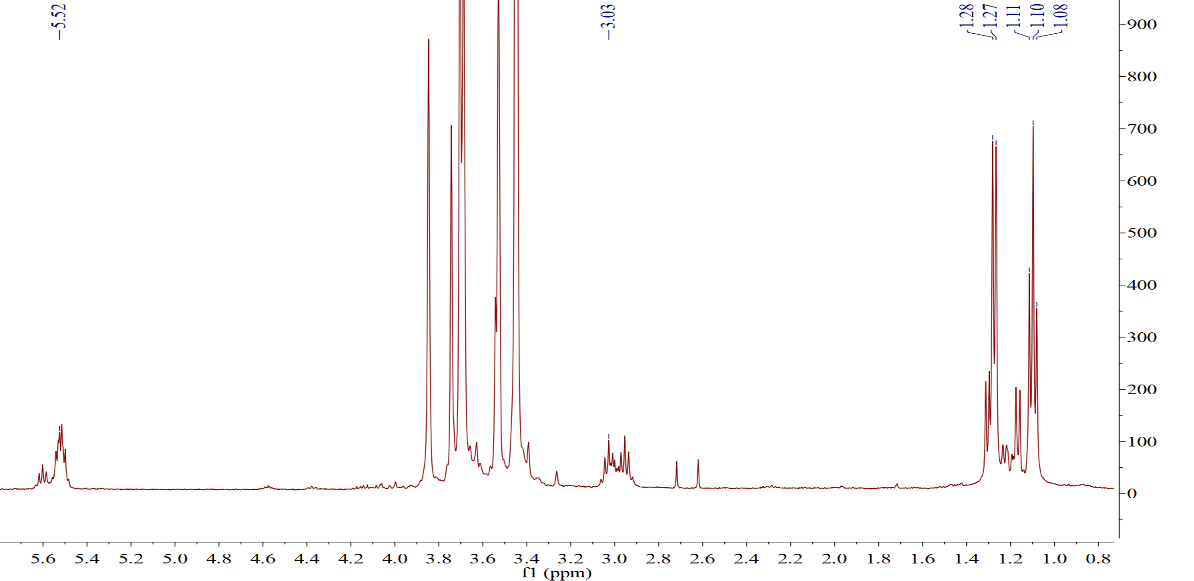

Supplement: Supplementary file 1 — Supplementary file1 (DOCX 1636 kb) [file 42995_2020_66_MOESM1_ESM.docx]
